# Expression profiles analysis reveals an integrated miRNA-lncRNA signature to predict survival in ovarian cancer patients with wild-type BRCA1/2

**DOI:** 10.18632/oncotarget.19590

**Published:** 2017-07-26

**Authors:** Liyuan Guo, Yan Peng, Yuanyuan Meng, Yunduo Liu, Shangshang Yang, Hong Jin, Qi Li

**Affiliations:** ^1^ Gynecological Cancer Department, Harbin Medical University Cancer Hospital, Harbin 150081, China; ^2^ Disease Prevention Center, First Affiliated Hospital of Heilongjiang University of Chinese Traditional Medicine, Harbin 150040, China

**Keywords:** BRCA1/2, ovarian cancer, microRNA, long non-coding RNA, prognosis

## Abstract

Emerging evidence shows that dysregulated expression of microRNAs (miRNAs) and long non-coding RNAs (lncRNAs) were closely linked with disease progression, including cancers. However, the joint predictive power of miRNAs and lncRNAs in prognosis for ovarian cancer (OV) patients with wild-type BRCA1/2 is as yet unknown. In this study, we sought to assess the joint predictive power of miRNAs and lncRNAs by integrating miRNA and lncRNA expression profiles and clinical data of 281 OV patients with wild-type BRCA1/2 from The Cancer Genome Atlas (TCGA) project. Finally, we identified an integrated miRNA-lncRNA signature composing of two lncRNAs (*LINC01234* and *CCDC144NL-AS1*) and two miRNAs (*miR-637* and *miR-129-5p*) which can effectively classify OV patients with wild-type BRCA1/2 into groups with the good and poor outcome. The prognostic value of the integrated miRNA-lncRNA signature was validated in the testing cohort and entire TCGA cohort. Multivariate analysis demonstrated the independence of the integrated miRNA-lncRNA signature of known other clinical factors. Further analysis suggested that patients who were in the low-risk group based on the signature achieved a better CR from platinum-based chemotherapy compared with patients in the high-risk group. Our results indicated that this integrated miRNA-lncRNA signature may have important clinical implications for risk stratification of ovarian cancer patients with wild-type BRCA1/2.

## INTRODUCTION

Ovarian cancer (OV) is one of the most malignant gynecological cancer and remains the most common cause of cancer-related deaths [1]. Ovarian cancer can be divided into two groups on the basis of genetic changes: low-grade tumors with mutations in KRAS and BRAF, genomic instability, and high-grade tumors (HGS-OV) with mutations in BRCA and TP53 [1, 2]. A conventional treatment regimen is tumor debulking followed by platinum-based chemotherapy and poly (ADP-ribose) polymerase (PARP) inhibitors [2]. OV patients carrying aberrations in BRCA1 or BRCA2 (BRCA1/2) have a better prognosis than those with wild-type BRCA1/2, because BRCA1/2 mutations are associated with response to platinum-based chemotherapy or PARP inhibitors [3, 4]. Emerging evidence indicated that a proportion of OV patients with wild-type BRCA1/2 may also harbor homologous recombination (HR) deficiency and have a favorable prognosis when subjected to platinum-based treatment [5]. Therefore, identification of BRCA1/2 alteration non-carriers benefiting from platinum-based chemotherapy is an important step toward achieving individual appropriate treatment strategies for serous ovarian carcinoma patients

Advances in genomic and transcriptomic technology and analysis have accelerated exponentially our understanding of human genome indicated that although at least 90% of the genome is actively transcribed, protein-coding genes only represent < 2% of the total genome sequence [6]. The human transcriptome was found to be composed of protein-coding genes and non-coding RNA (ncRNA) [7, 8]. ncRNA can be classified into two major classes: small ncRNAs (<200bp) and long non-coding RNAs (lncRNAs, >200bp). MicroRNAs (miRNAs) are a big class of small ncRNAs and are transcribed as primary miRNA precursors. miRNA can exert the regulatory function at the posttranscriptional level by binding to the 3’UTRs of the target mRNA through base pairing [9]. lncRNAs, a newly discovered class of ncRNAs, were defined as lncRNAs as RNA molecules that may function as either primary or spliced transcripts and do not fit into known classes of small RNAs [10]. Many computational methods and tools have been proposed and widely used for predicting and analyzing ncRNAs, such as 2L-piRNA, repRNA/repDNA, Pse-in-One [11–13].

Increasing evidence suggests that miRNAs and lncRNAs play crucial roles in a variety of biological processes, such as the proliferation, development and differentiation [14, 15]. Emerging evidence also shows that miRNAs and lncRNAs constitute an important component of disease biology and their dysregulated expression was closely associated with disease progression, including cancers [16–19]. Previous studies have demonstrated that miRNAs and lncRNAs can serve as stable biomarkers for cancer detection, diagnosis, and prognosis assessment [20–36].

Despite the fact that some miRNAs or lncRNAs-related signatures have been proposed in OV and their prognostic value is being investigated [24, 25, 33, 37–39], the joint predictive power of miRNAs and lncRNAs in prognosis for patients with wild-type BRCA1/2 is still unknown. In this study, we sought to assess the joint predictive power of miRNAs and lncRNAs by integrating miRNA and lncRNA expression profiles and clinical data of 281 OV patients with wild-type BRCA1/2 from The Cancer Genome Atlas (TCGA) project.

## RESULTS

### Identification of prognostic lncRNAs and miRNAs in OV patients with wild-type BRCA1/2

To identify prognostic lncRNAs and miRNAs in OV patients with wild-type BRCA1/2, we performed univariate and multivariate Cox regression analysis for expression data of each lncRNA and miRNA with OS as the dependent variable. A total of four genes, including two lncRNAs and two miRNAs, was selected as prognostic factors that are independently significantly associated with OS (p<0.05). As shown in Table 1, three prognostic factors (LINC01234, CCDC144NL-AS1 and miR-129-5p) have positive coefficient, indicating these three prognostic genes may be risk factors and their over-expression is associated with shorter OS, whereas the remaining prognostic miRNA (miR-637) with negative coefficients may be protected factors and its over-expression is associated with longer OS.

**Table 1:** Two lncRNAs and two miRNAs that are independently significantly associated with overall survival

Gene symbol	Genomic coordinates (GRCH 38)	P-value^a^	Hazard ratio^a^	Coefficient^a^
LINC01234	Chr 12: 113679459-113773683(−)	0.002	1.431	0.359
CCDC144NL-AS1	Chr 17: 20868433-21002276(+)	0.042	1.235	0.211
miR-129-5p	chr7: 128207872-128207943(+)	0.034	1.421	0.351
MIR637	chr19: 3961414-3961512(−)	0.039	0.792	−0.234

^a^Derived from univariate Cox regression analysis.

### Development of an integrated miRNA-lncRNA signature in the discovery cohort

To develop an integrated miRNA-lncRNA signature in survival prediction for patients with wild-type BRCA1/2, we performed multivariate Cox regression analysis on the expression data of four prognostic lncRNAs in the discovery cohort and miRNAs with OS as a dependent variable and other individual clinical features as explanatory variables to consider the mutual effect among factors. Then an integrated miRNA-lncRNA signature was constructed using risk score method based on the expression of prognostic miRNAs and lncRNAs and their regression coefficient in the above multivariate Cox regression as previously described [28]: Risk Score = ((0.4222 × expression value of LINC01234)+(−0.0341 × expression value of CCDC144NL-AS1) +(0.4461 × expression value of miR-129-5p)+(−0.2265 × expression value of miR-637)). With the integrated miRNA-lncRNA signature, we calculated the risk score for each patient in the discovery cohort. Patients of discovery cohort were ranked according to their risk scores. Using the median risk score of the discovery cohort as high-risk cutoff value, a patient was considered as high-risk if risk score is higher than the cutoff value and as low-risk if not.

**Figure 1:**
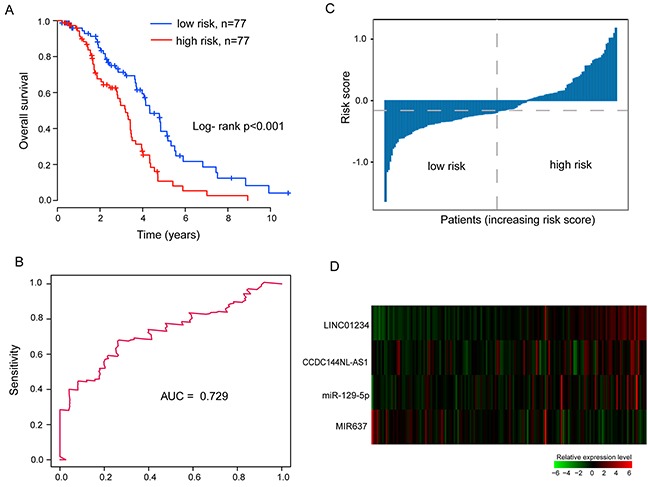
Association between the integrated miRNA-lncRNA signature and overall survival of ovarian cancer patients with wild-type BRCA1/2 in the discovery cohort **(A)** Kaplan-Meier curves of overall survival in high-risk group and low-risk group. **(B)** Time-dependent ROC curve analysis of the survival prediction based on the risk score with five years as the time point. **(C)** Distribution of risk scores of patients in the discovery cohort. **(D)** Expression heat map of four prognostic factors in the discovery cohort.

Finally, patients of discovery cohort were grouped into the high-risk group (n=77) and low-risk group (n=77). Analysis of Kaplan-Meier survival curves and log-rank test demonstrated the significant difference in OS between high-risk group and low-risk group (p<0.001, log-rank test) (Figure 1A). Patients with high-risk scores had a shorter median survival than those with low-risk scores (median survival 3.2 years vs.4.34 years) (Table 2). The 3- and 5-year survival rate of the high-risk group were 52.8% and 10.7%, respectively, whereas the corresponding rates in the low-risk group were 71.3% and 38.4%, respectively (Table 2). Detailed information for OS in the discovery cohort was 69.3% at 2-years, 27.4% at 4-years and 5.4% at 6-years in the low-risk group, versus 84.9%, 59.3% and 21.7% respectively in the high-risk group (Table 2).

**Table 2:** Kaplan-Meier survival curves analysis for overall survival in the discovery cohort, testing cohort and entire patient cohort

Patient cohort	Deaths/patient (%)	Median survival (years, 95% CI)	Survival rate (%)				
			**2 year**	**3 year**	**4 year**	**5 year**	**6 year**
Discovery cohort							
High-risk (n=77)	53/77 (68.8)	3.2 (2.78-3.5)	69.3	52.8	27.4	10.7	5.4
Low-risk (n=77)	44/77 (57.1)	4.34(3.98-5.33)	84.9	71.3	59.3	38.4	21.7
Testing cohort							
High-risk (n=76)	53/76 (69.7)	3.17(2.87-3.78)	81.3	59.3	30.7	18.4	16.1
Low-risk (n=51)	25/51 (49)	4.07(3.71-NA)	86.5	67.9	55.7	38	34.2
Entire cohort							
High-risk (n=153)	106/153(69.3)	3.17(2.87-3.5)	74.8	56.2	29.2	14.9	11.2
Low-risk (n=128)	69/128(53.9)	4.15(3.98-5.14)	85.6	69.4	57.9	38.4	25.9

The prognosis prediction capability of the integrated miRNA-lncRNA signature was further evaluated by Time-dependent ROC which achieved an AUC value of 0.729 at 5-years of OS (Figure 1B). Figure 1C and 1D shows the distribution of patient risk scores and expression level of prognostic lncRNAs and miRNAs, ranked according to the risk scores for the integrated miRNA-lncRNA signature. Among four genes, three (*LINC01234*, *CCDC144NL-AS1* and *miR-129-5p*) were shown to be protective factors and were associated with high risk, and the remaining one miRNA (miR-637) was shown to be a risk factor and was associated with low-risk. As shown in Figure 1C, patients with high-risk scores tended to express high-risk lncRNAs and miRNAs, whereas patients with low-risk scores tended to express protective miRNA.

### Validation of the integrated miRNA-lncRNA signature in the testing cohort

**Figure 2:**
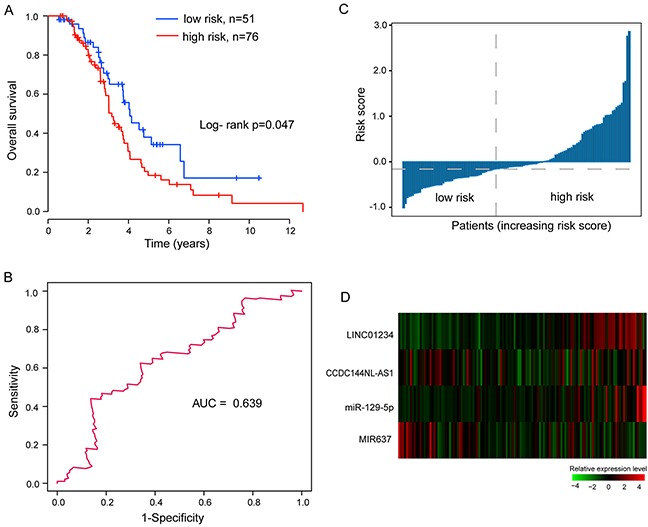
Association between the integrated miRNA-lncRNA signature and overall survival of ovarian cancer patients with wild-type BRCA1/2 in the testing cohort **(A)** Kaplan-Meier curves of overall survival in high-risk group and low-risk group. **(B)** Time-dependent ROC curve analysis of the survival prediction based on the risk score with five years as the time point. **(C)** Distribution of risk scores of patients in the testing cohort. **(D)** Expression heat map of four prognostic factors in the testing cohort.

To confirm the power of the integrated miRNA-lncRNA signature in survival prediction in patients with wild-type BRCA1/2, the integrated miRNA-lncRNA signature was validated in 127 patients of the testing cohort. We first calculated the risk score for 127 patients in the testing cohort using the same risk score model. By using the same high-risk cutoff value from discovery cohort, patients of the testing cohort were classified into the high-risk group (n=76) and low-risk group (n=51). As in the discovery group, patients in the high-risk group had significantly shorter overall survival than those in the low-risk group (median survival 3.17 years vs. 4.07 years, p=0.047, log-rank test) (Table 2) (Figure 2A). The 3- and 5-year survival rate of the high-risk group were 59.3% and 18.4%, respectively, whereas the corresponding rates in the low-risk group were 67.9% and 38%, respectively (Table 2). Detailed information for OS in the testing cohort was shown in Table 2. Overall survival was 81.3% at 2-years, 30.7% at 4-years and 16.1% at 6-years in the high-risk group, versus 86.5%, 55.7% and 34.2% respectively in the low-risk group (Table 2).

Time-dependent ROC curves were used to assess the prognostic power of the integrated miRNA-lncRNA signature. The AUC for the integrated miRNA-lncRNA signature was 0.639 at 5-years of OS (Figure 2B). We also showed the distribution of patient risk scores and expression level of prognostic lncRNAs and miRNAs, ranked according to the risk scores for the integrated miRNA-lncRNA signature (Figure 2C and 2D). Similar to the discovery cohort, patients with high-risk scores tended to express high levels of two risky lncRNAs and one miRNAs, whereas patients with low-risk scores tended to express high levels of protective miRNA.

### Further validation of the integrated miRNA-lncRNA signature in the entire patient cohort

**Figure 3:**
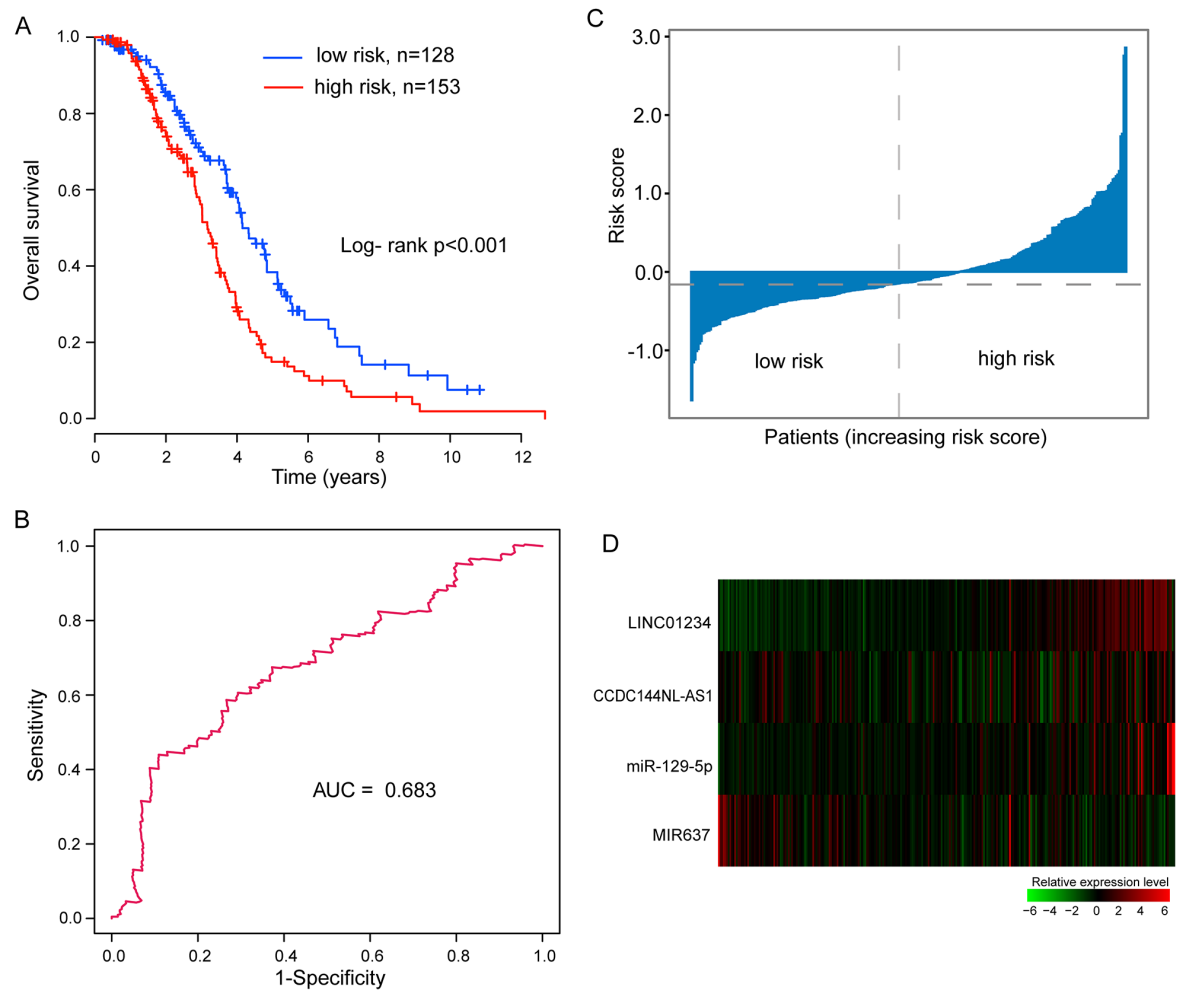
Association between the integrated miRNA-lncRNA signature and overall survival of ovarian cancer patients with wild-type BRCA1/2 in the entire patient cohort **(A)** Kaplan-Meier curves of overall survival in high-risk group and low-risk group. **(B)** Time-dependent ROC curve analysis of the survival prediction based on the risk score with five years as the time point. **(C)** Distribution of risk scores of patients in the entire patient cohort. **(D)** Expression heat map of four prognostic factors in the entire patient cohort.

Additional investigation of the integrated miRNA-lncRNA signature in survival prediction was performed in the entire patient cohort. The same risk score model and cutoff value as those derived from the discovery cohort classified 281 patients of the entire patient cohort into the high-risk group (n=153) and low-risk group (n=128), respectively. Consistent with the discovery and testing cohorts, The differences of survival curves between high-risk group and low-risk group were statistically significant (Figure 3A). The patients in the low-risk group also had a better median survival than those in the high-risk group (median survival 4.15 years vs. 3.17 years, p<0.001, log-rank test) (Table 2), as shown in Figure 3A. The 3- and 5-year survival rate of the high-risk group were 56.2% and 14.9%, respectively, whereas the corresponding rates in the low-risk group were 69.4% and 38.4%, respectively (Table 2). Detailed information for OS in the entire patient cohort was shown in Table 2. Overall survival was 74.8% at 2-years, 29.2% at 4-years and 11.2% at 6-years in the high-risk group, versus 85.6%, 57.9% and 25.9% respectively in the low-risk group (Table 2).

The AUC of time-dependent ROC curves for the risk score was 0.683 at five years of OS (Figure 3B). The distribution of patient risk scores and expression level of prognostic lncRNAs and miRNAs, ranked according to the risk scores for the integrated miRNA-lncRNA signature were shown in Figure 3C and 3D and the results were similar to those observed in the discovery dataset and testing dataset above

### Independence of the integrated miRNA-lncRNA signature and other clinical factors

To assess whether the integrated miRNA-lncRNA signature was independent of other clinical factors, univariate and multivariate Cox regression analyses were conducted in the entire patient cohort including risk scores, age, tumor stage and tumor grade. As shown in Table 3, univariate analysis revealed that the risk score and age were both significantly associated with OS in OV patients. Moreover, the risk score still has a significantly associated with OS after adjusted by other clinical factors (p<0.001) (Table 3). These Cox results demonstrated that the prognostic value of the integrated miRNA-lncRNA signature is independent of other clinical factors for survival prediction of OV patients with wild-type BRCA1/2.

**Table 3:** Univariate and multivariate Cox regression analysis

Variables	Univariate analysis			Multivariate analysis		
	HR	95%CI	P value	HR	95%CI	P value
Risk score	1.834	1.343-2.505	<0.001	1.791	1.307-2.456	<0.001
Age	1.022	1.007-1.037	0.004	1.018	1.003-1.033	0.018
Stage	1.153	0.835-1.591	0.388	1.14	0.812-1.602	0.449
Grade	1.351	0.887-2.058	0.16	1.303	0.852-1.993	0.222

### The association between the integrated miRNA-lncRNA signature and complete response

**Figure 4:**
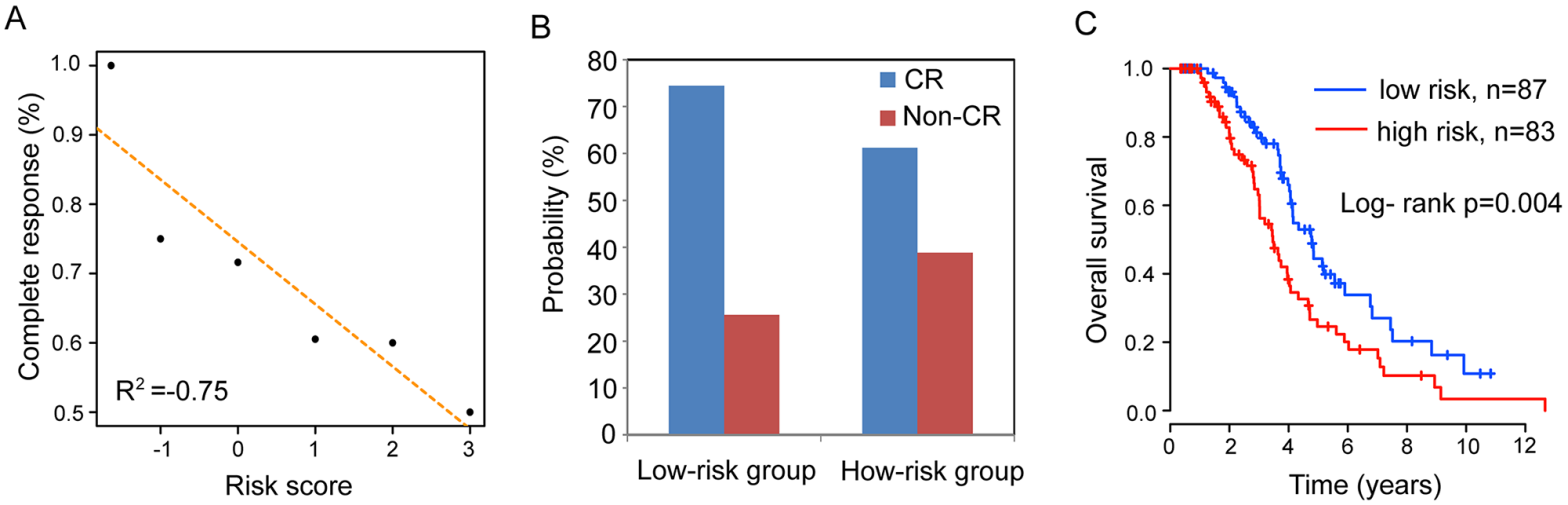
The association between risk score and complete response **(A)** Correlation of risk score with complete response. **(B)** Differences in complete response ratios between high-risk group and low-risk group. **(C)** Kaplan-Meier curves of overall survival in high-risk group and low-risk group of OV patients achieving complete response.

In order to examine the association between the integrated miRNA-lncRNA signature and complete response (CR), we plotted the percentage of OV patients achieving CR as a function of the risk score according to previous study [25], and observed a close association between the integrated miRNA-lncRNA signature and complete response. (Pearson correlation coefficient r^2^=-0.75) (Figure 4A). Patients with low-risk scores tended to have a high likelihood of CR and those with high-risk scores had a low likelihood of CR. In the high-risk group, 61.2% of patients achieved CR, whereas 74.4% of patients in the low-risk group achieved CR (Fisher exact test, p=0.03) (Figure 4B). Moreover, we found that the integrated miRNA-lncRNA signature could further subdivide OV patients achieving CR into the high-risk group and low-risk group with significantly different survival times (Figure 4C). Patients achieving CR in the high-risk group had a significantly shorter median OS than did those in the high-risk group (median survival 3.47 years vs. 4.78 years, p=0.004, log-rank test) (Figure 4C). The 5-year survival rate of the high-risk group was 25.5%, whereas the corresponding rate in the low-risk group was 44.4%.

## DISCUSSION

Although tumor debulking followed by platinum-based chemotherapy and poly (ADP-ribose) polymerase (PARP) inhibitors have improved OV patients’ survival, most patients will face the high rate of relapse resulting in a 5-year survival rate less than 40% [40]. About 30% of ovarian cancer patients are found to be platinum resistant only after having undergone multiple cycles of nonbeneficial, toxic chemotherapy [41]. It is well known that BRCA1 and BRCA2 mutations are one of the known risk determinants for the development of ovarian carcinoma and BRCA1/2 mutations is associated with response to platinum-based chemotherapy [3]. Emerging evidence has suggested that a proportion of OV patients with wild-type BRCA1/2 may have the favorable prognosis when subjected to platinum-based treatment [5, 41]. Therefore, it is critical to identify BRCA1/2 alteration non-carriers benefiting from platinum-based chemotherapy.

With the data availability of microarray-based expression and RNA-Seq-based transcriptomics, gene expression-based signature has been widely used for cancer detection, diagnosis, and prognosis assessment. Several mRNA-based prognostic signatures have been developed in previous studies. For example, Spentzos identified an independent 115-gene signature referred to as the Ovarian Cancer Prognostic Profile [42]. Another study by Hao *et al*. proposed a homologous recombination deficiency-derived 116 genes to predict outcome and response to platinum therapy [41]. Increasing evidence indicates that miRNAs and lncRNAs may have greater utility than mRNAs as prognostic markers because of intrinsic advantages over mRNAs. For example, mRNA levels are only indirectly indicative of the levels of the functional product of coding genes, whereas expression of miRNAs and lncRNAs is a direct indicator of the tumor status [43]. Identifying miRNA or lncRNA-based signature is of great interest in recent times. Gu *et al.* used TCGA data to investigate the association between miRNA deregulation and outcome of patients with wild-type BRCA1/2 and identified that the three-miRNA signature (hsa-miR-146a, hsa-miR-148a and hsa-miR-545), which target BRCA1/2, in patients with wild-type BRCA1/2 was associated with good outcome [5]. Although a few recent study on the prognostic value of lncRNAs or miRNAs in OV, the possibility of the prognostic value of the integrated miRNA and lncRNA signature for OV patients with wild-type BRCA1/2 has not been investigated systematically.

In this study, using genome-wide lncRNA and miRNA expression profiles and clinical data in a large number of OV patients with wild-type BRCA1/2 from TCGA, we investigated the prognostic value of the integrated miRNA and lncRNA signature in survival prediction for OV patients with wild-type BRCA1/2. By performing univariate and multivariate Cox regression analysis for miRNA and lncRNA expression data, we identified an integrated miRNA-lncRNA signature composing of two lncRNAs (LINC01234, CCDC144NL-AS1) and two miRNAs (miR-637 and miR-129-5p). Of them, three prognostic factors (*LINC01234*, *CCDC144NL-AS1* and *miR-129-5p*) are risk factors and the remaining prognostic miRNA (miR-637) is protected factors. Then a linear combination of these four prognostic factors was constructed and as an integrated miRNA-lncRNA signature for survival prediction of patients with wild-type BRCA1/2. To our knowledge, this is the first report of an integrated miRNA-lncRNA expression signature predicting OV patient survival. By applying the integrated miRNA-lncRNA expression signature to the discovery cohort, testing cohort and entire TCGA cohort, a clear separation was observed in the survival curves between high-risk group and low-risk group, suggesting the good reproducibility of this integrated miRNA-lncRNA signature for survival prediction of patients with wild-type BRCA1/2. Patients with low-risk scores tended to have a better outcome, whereas patients with low–risk scores tended to have a poor outcome. Moreover, the integrated miRNA-lncRNA signature is independent of known other clinical factors. Further analysis suggested that patients who were in the low-risk group based on the signature achieved a better CR from taxane-based chemotherapy compared with patients in the high-risk group.

Of the four prognostic factors included in the integrated miRNA-lncRNA signature, two miRNAs have been well functionally characterized in previous studies. These two miRNAs have been found to be associated with many cancers. MiR-637 have been reported as a tumor suppressor and involved in the differentiation of human mesenchymal stem cells [44], which is consistent with our research. Zhang *et al.* found that miR-637 was down-regulated in ovarian cancer cell lines compared to IOSE [45]. Another study performed by Tan et al. reveal the functional role of miR-129-5p in inhibiting ovarian cancer cell proliferation and survival via direct suppression of transcriptional co-activators YAP and TAZ [46]. However, functional roles of two lncRNAs in this integrated miRNA-lncRNA signature have been reported in previous studies yet, and deserve further studied in the further studies.

In conclusion, we have identified an integrated miRNA-lncRNA signature by analyzing genome-wide miRNA and lncRNA expression profiles which can effectively classify OV patients with wild-type BRCA1/2 into groups with the good and poor outcome. This integrated miRNA-lncRNA signature showed independence of other clinical factors. However, independent validation and the functional elucidation of this integrated miRNA-lncRNA signature is also required.

## MATERIALS AND METHODS

### Patient dataset and clinical information

Clinical and pathological information of OV patients with wild-type BRCA1/2 were obtained from The Cancer Genome Atlas (TCGA) project (https://cancergenome.nih.gov/). The somatic and germline mutation information of BRCA1 and BRCA2 genes were obtained from the cBioPortal Cancer Genomics (http://www.cbioportal.org/). Finally, 281 wild-type BRCA1/2 OV patients receiving a platinum regimen was analyzed in our study. All 281 OV patients with wild-type BRCA1/2 were separated into the discovery cohort including 154 patients and testing cohort including 127 patients according to samples batch information.

### Genome-wide miRNA and lncRNA expression profiles of OV patients with wild-type BRCA1/2

We acquired the genome-wide miRNA and lncRNA expression profiles data from a previously published study by Du *et al.*, which gave rise to a total of 15857 lncRNA genes by repurposing data from TCGA Affymetrix Human Exon 1.0 ST array [47]. The level 3 miRNA expression profile based on the Agilent 8 × 15 K Human microRNA-specific microarray V2 platform was obtained from TCGA data portal.

### Identification of an integrated miRNA-lncRNA signature

The univariate and multivariate Cox regression analysis was used to identify prognostic lncRNAs and miRNAs that significantly associated with OS in the discovery cohort. Those miRNAs and lncRNAs with p < 0.05 after univariate Cox regression analysis followed by multivariate Cox regression analysis was selected as prognostic miRNAs and lncRNAs. Then these prognostic miRNAs and lncRNAs with clinical variables (including age, grade, stage and tumor size) were subject to multivariate Cox regression model to obtain their relative predictive power for OS in the discovery cohort. Finally, an integrated miRNA-lncRNA signature was developed by linear combination of the expression values of prognostic miRNAs and lncRNAs and the Cox regression coefficient as the weight in the discovery cohort. Each OV patient with wild-type BRCA1/2 was assigned a risk score according to the integrated miRNA-lncRNA signature and predicted as high-risk patient or low-risk patient using the median risk score as the cutoff point.

### Statistical analysis

Kaplan-Meier survival curves were used to estimate OS for patients in the low- and high-risk groups, and the log-rank test was used to assess the differences in OS time between the high-risk and low-risk patients. Time-dependent receiver operating characteristic (ROC) curve analysis with five years as the time point was used to compare the sensitivity and specificity of the OS prediction based on the integrated miRNA-lncRNA signature. Multivariate analyses were performed using Cox proportional hazards regression model to determine whether the integrated miRNA-lncRNA signature independent of other clinical variables, such as age, grade and so on. The hazard ratio (HR) and 95% confidence intervals (CI) were estimated by Cox proportional hazards regression model. All analyses were performed using the R/Bio-Conductor (version 3.0.2): survival curves using R package “survival” [48], and time-dependent ROC analysis using R package “survivalROC” [49].
